# Effects of Soccer Boot Insoles on Muscle Activity and Ankle Inversion Proprioception in Drop Landing Tasks in Patients with and without Chronic Ankle Instability

**DOI:** 10.5114/jhk/205685

**Published:** 2025-10-24

**Authors:** Ratakorn Aimkosa, Oren Tirosh, Charlotte Genderton, Doa El-Ansary, Roger Adams, Jia Han

**Affiliations:** 1 School of Exercise and Health, Shanghai University of Sport, Shanghai, China.; 2 School of Health Science, Mae Fah Luang University, Chaing Rai, Thailand.; 3 School of Health and Biomedical Sciences, Royal Melbourne Institute of Technology University, Melbourne, Australia.; 4 Research Institute for Sport and Exercise, University of Canberra, Canberra, ACT, Australia.; 5 College of Rehabilitation Sciences, Shanghai University of Medicine and Health Science, Shanghai, China.

**Keywords:** ankle injuries, electromyography, foot orthoses

## Abstract

Chronic ankle instability (CAI) poses challenges in sports. Textured insoles are a promising intervention for optimizing ankle muscle activity and enhancing ankle proprioceptive ability in individuals with CAI. This study investigated the effects of different types of insoles on ankle muscle activity and ankle inversion proprioception during drop-landing tasks. Thirty-two soccer players (16 CAI, 16 non-CAI) participated in the study. Electromyographic (EMG) activity of the tibialis anterior (TA), medial gastrocnemius (MG), and peroneus longus (PL) muscles was recorded during drop-landing tasks using the ankle inversion discrimination apparatus for landing (AIDAL) with textured insoles (TI) and standard flat insoles (SI). Ankle inversion proprioception was assessed using the AIDAL. Muscle activity was analyzed for pre- and initial contact phases. CAI players showed significantly reduced TA activity compared to non-CAI players during both pre-initial (2.62 ± 1.86 vs. 5.53 ± 5.15; p = 0.04) and initial contact phases (18.7 ± 13.6 vs. 29.9 ± 20.6; p = 0.01). CAI players also exhibited greater MG activity during pre-initial (16 ± 6.15 vs. 12.5 ± 6.38; p = 0.03) and initial contact phases (60.8 ± 32.5 vs. 41.5 ± 14.8; p = 0.004), and higher PL activity during the pre-initial contact phase (7.39 ± 4.75 vs. 4.28 ± 2.83; p = 0.003). No significant CAI/insole main effects or interactions (p > 0.05) were observed in AIDAL proprioceptive scores. Overall, CAI players showed significant alterations in ankle muscle activity during drop-landing tasks. Increased PL activity during the pre-initial contact phase may serve as a compensatory strategy to enhance ankle stability, while reduced TA and increased MG activation during pre-initial and initial contact phases may contribute to the high incidence of recurrent ankle sprains in CAI individuals. Additionally, the lack of immediate effects from insoles on muscle activity suggests that their influence on sensory input and foot positioning may require prolonged use to induce measurable neuromuscular adaptations.

## Introduction

Soccer is a popular sport requiring players to frequently change direction, sprint, and jump, necessitating a consistent functional stability of the ankle joint to execute these demanding movements effectively (Cortis et al., 2013). These compound movement tasks place significant physical demands on the lower limbs, often leading to injuries, with the ankle being the most affected area (Walls et al., 2016). According to the data from a retrospective study, the mean incidence of ankle injuries was one per player per year (Cloke et al., 2009). Other findings suggest that soccer players experience a higher incidence of ankle injuries compared to athletes in other sports (Bailey and Firth, 2017). Lateral ankle sprains are among the most prevalent lower limb injuries in soccer (Gribble et al., 2016). This condition may result in damage to both soft and bony tissue, leading to pain and functional deficits in the ankle joint (Hertel, 2002). Following lateral ankle sprains, athletes that have not fully recovered or have had inadequate treatment are more susceptible to developing long-term symptoms, which can lead to chronic ankle instability (Delahunt and Remus, 2019; Hertel and Corbett, 2019).

Chronic ankle instability (CAI) is a condition marked by recurrent ankle sprains and a persistent sensation of the ankle giving way. This instability often leads to diminished athletic performance and is accompanied by enduring symptoms such as pain, weakness, reduced range of motion, and impaired functionality of the ankle joint (Hertel and Corbett, 2019; Luan et al., 2024). This condition is characterized by significant neuromuscular impairment, including proprioceptive deficits and muscle activation dysfunction. A systematic review of existing research highlights that individual with CAI exhibit significant impaired ankle proprioception (Xue et al., 2021). Additionally, research assessing proprioception under functional conditions, such as standing, walking, and landing, confirms these findings. For instance, during standing tasks, individuals with CAI demonstrate greater postural sway and instability, indicating compromised proprioceptive control (Xue et al., 2023). Similarly, assessments conducted during walking reveal that CAI individuals exhibit altered gait mechanics, reflecting impaired proprioceptive feedback and motor control (Lee et al., 2021). During landing tasks, individuals with CAI exhibit significantly worse ankle inversion proprioceptive performance compared to their non-CAI counterparts. This impairment reflects a delayed and less accurate response to changes in the ankle position during dynamic activities, possibly resulting in improper foot positioning upon impact (Kang et al., 2022; You and Huang, 2022).

Alterations to muscle activation patterns have been identified in lower limb muscle activity among individuals with CAI when compared with people without CAI. Specifically, studies have found that individuals with CAI exhibited longer latency in the activation of the peroneus longus and peroneus brevis muscles (Simpson et al., 2019). Additionally, there was a decrease noted in peroneus longus, and an increase in tibialis anterior muscle activity, during the pre-initial contact phase of single leg drop-landing tasks compared to non-CAI counterparts (Tretriluxana et al., 2021). In contrast, others have found that individuals with CAI demonstrate higher activity levels in the peroneus longus and gluteus maximus muscles compared to non-CAI participants in the pre-landing phase of the lateral hop task (Webster et al., 2016). These changes in muscle activity may indicate a compensation mechanism that is crucial for stabilizing the joints during dynamic movements. However, these variations might potentially impair the stability of the joint and increase the risk of injury. Therefore, interventions emphasizing the enhancement of ankle proprioception and the optimization of muscle activity are crucial for the efficient management of CAI.

In recent years, there has been increasing interest in interventions designed to manage the CAI by stimulating the cutaneous mechanoreceptors on the plantar surface of the foot through various types of foot orthoses (Abbasi et al., 2019; Palomo-Fernández et al., 2023). The application of shoe insoles has emerged as a potential strategy to enhance ankle proprioception, particularly as measured during standing (Steinberg et al., 2015, 2016a). These insoles are designed to stimulate the cutaneous mechanoreceptors on the plantar surface of the foot, thereby providing additional sensory feedback that can improve awareness of the ankle position. While previous studies have shown the positive effects of the application of insoles on ankle proprioception during static tasks, the effects of different types of insoles on ankle proprioception and lower limb muscle activities in individuals with CAI during dynamic tasks such as drop-landing remain insufficiently understood. Therefore, analyzing neuromuscular control in individuals with CAI during dynamic tasks is essential for gaining comprehensive insight into how different insoles can influence ankle stability and muscle function, which may ultimately lead to more effective interventions for managing CAI.

Therefore, the purpose of this study was to investigate the effects of different types of insoles, specifically standard flat insoles (SI) and textured insoles (TI), on lower limb muscle activity and ankle inversion proprioception during drop-landing tasks in soccer players with and without CAI. It was hypothesized that soccer players with CAI would exhibit distinct muscle activity patterns and proprioceptive performance when using different types of insoles.

## Methods

### Participants

Thirty-two university soccer players with or without CAI (16 for each group) participated in this study. All the participants were currently engaged in either competitive or recreational athletic activity. The following criteria were used to determine eligibility for CAI participants: (i) a history of at least one significant lateral ankle sprain, with the initial sprain occurring at least 12 months earlier and the most recent sprain occurring at least three months before the study enrollment; (ii) episodes of the ankle joint giving way and feelings of instability; and (iii) Cumberland Ankle Instability Tools (CAIT) score less than 25 (Wright et al., 2014). The inclusion criteria for the non-CAI participants were as follows: (i) no symptoms of an ankle injury; (ii) a score equal to or greater than 25 points on the CAIT. The exclusion criteria for all groups included: (i) a history of previous injuries to the musculoskeletal structures (i.e., bones, joint structure, nerves) of the lower limb; (ii) a history of surgery or fracture to either lower limb that required realignment; or (iii) any acute injuries that occurred within the previous three months prior to the study enrollment. Before data collection, each participant read and signed an informed consent document. This study was approved by the ethics committee of the Mae Fah Luang University, Chaing Rai, Thailand (protocol code: EC 20189-18; approval date: 21 January 2021).

### Instruments

#### Electromyography

The Trigno^TM^ wireless surface EMG system (Delsys, Wireless Biofeedback System, USA) was used to record muscle activity during drop-landing task performance on the AIDAL at pre-initial contact and initial contact phases. Each sensor measured 37 x 26 x 15 mm, weighed 14 g, and comprised two bar electrodes and two reference bar electrodes. According to the European Recommendation for Surface Electromyography (SENIAM) (Hermens et al., 2000), three EMG electrodes were positioned on participants' testing legs over the most prominent part of the muscle belly of the TA, MG, and PL muscles. Prior to electrode placement, each site was shaved and cleaned with alcohol. The EMGworks Acquisition Program (Delsys) was used to assess the signal quality prior to data collection. All EMG data were recorded at a sampling frequency of 1926 Hz.

#### Force Sensitive Resistor

To determine foot contact time, a customized Force Sensitive Resistor (FSR) plate with a customized landing surface was integrated and synchronized with the Delsys EMG system. This device consisted of the FSR sensor placed between two metal plates. The FSR sensor ranged from 0 to 100 N, and had sensitivity of 15 ± 5 g, and a response time of 0.5–1 ɥs. It was powered by a micro-USB cable and regulated by an Arduino IDE module operating at 2.67 MHz.

#### Ankle Inversion Discrimination for Landing

**Figure 1:**
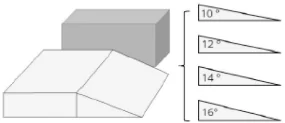
Ankle inversion discrimination apparatus for landing (AIDAL).

The ankle inversion discrimination apparatus for landing (AIDAL) provides discrimination scores that reflect participants' sensitivity to minor changes in ankle inversion extent (Han et al., 2021). This device consists of three parts: (i) a take-off platform; (ii) a horizontal supporting platform; and (iii) an inclined landing platform for the testing foot, which can be set at four different angles (Figure 1). To ensure lower limb muscle pre-activation, a 10-cm difference between the take-off and the horizontal landing platforms was employed (Han et al., 2021). The AIDAL provides ankle proprioceptive discrimination scores by assessing participants’ ability to distinguish between four different angles of the ankle inversion tilt as they perform drop-landing tasks from the take-off platform onto four predetermined inclined landing platforms. The four possible inclined platforms were 10°, 12°, 14°, and 16°. Modified surfaces were applied to all the AIDAL platforms to increase the ecological validity of the ankle proprioception test.

#### Insoles

**Figure 2:**
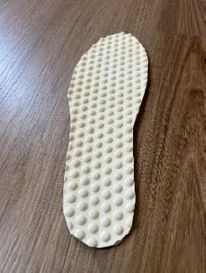
Characteristics of the textured insoles used in this study.

The textured insoles (TI) that were used for this study had a thickness of 2 mm and were made of an Ethylene Vinyl Acetate (EVA). The surface of the TI was made up of nodules that had a diameter of 7 mm and a height of 2 mm, and they were dispersed uniformly over the insole surface (Figure 2). Additionally, standard flat insoles (SI), which were made of the same EVA material but without nodules, were provided to participants as a control condition. Each participant in both groups was provided with pairs of both TI and SI, which were cut to fit and used as alternatives to the insoles in their soccer boots.

### Measures

During drop landing tasks, the EMG amplitudes recorded during each drop-landing task underwent specific signal processing. The recorded signals were filtered using a 10–500 bandpass filter, then rectified and smoothed using a root mean square technique within the EMG analysis software (EMGworks® Analysis version 4.7.9). The amplitude of each muscle was collected at two different time points: (i) 200 ms before the initial contact and (ii) at the initial contact (Simpson et al., 2019; Terada and Gribble, 2015). The initial contact time was identified by the FSR plate that detected when the vertical ground reaction force surpassed 15 N, indicating the occurrence of the initial contact (Kristianslund et al., 2011; Terada and Gribble, 2015). For data analysis, the muscle amplitudes at specific time points were normalized to each individual’s MVICs and expressed as a percentage (%MVICs) (Tretriluxana et al., 2021). These normalized values were subsequently utilized for further statistical analysis.

To obtain ankle inversion discrimination scores (AIDAL scores), raw response data were entered into four-by-four confusion matrices, which indicated the frequency with which each position number was produced for each degree of the ankle inversion tilt. Pair-wise receiver operator characteristic (ROC) curves were then generated using nonparametric signal detection techniques (Han et al., 2013), and the mean area under the curve (AUC) was computed to provide each participant with an ankle inversion discrimination score (Symes et al., 2010). The mean AUC values can range from 0.5, which corresponds to a random response, to 1.0, which demonstrates flawless discrimination between the four separate movement extents (Han et al., 2013).

### Design and Procedures

Prior to testing sessions, participants were given medical screening protocols and asked to complete the Cumberland Ankle Instability Tool (CAIT). For the testing sessions, the ankle with the lower CAIT scores of both CAI and non-CAI participants was selected as the testing leg. Participants then warmed up for 5 minutes by walking on a treadmill. Next, EMG electrodes were attached to the TA, MG, and PL muscles. Each participant engaged in three maximum voluntary isometric contractions (MVICs) for each muscle, against manual resistance. For the TA, participants lay in a supine position and maximally dorsiflexed the ankle joint against resistance. For the MG, participants lay on their stomach, with the knee stretched, and feet protruding over the edge of the bed. Participants subsequently maximally plantarflexed their foot against resistance. Finally, for the PL, participants sat on a bed with a support under the knee joint, then maximally plantarflexed and everted their foot against resistance. Following that, participants wore standard athletic socks, consisting of 89% polyamide and 11% spandex, and their soccer boots during the entire testing process, with the insoles (TI or SI) that had been randomly selected.

During the drop-landing tasks on the AIDAL, participants were directed to stand on the take-off platform with their non-testing leg while relaxing their testing leg over the edge of the platform. Participants then conducted a drop-landing, with their testing leg landing initially on the inclined platform and their non-testing leg landing simultaneously on the horizontal supporting platform, with their hips and knees naturally flexed. Participants then judged the angle level by responding with a number that best characterized it. The researcher recorded the response of participants and switched the inclined landing platform for the next trial. During the testing session, participants were required to keep their eyes forward to avoid visual information about the landing platform. The testing procedure comprised 12 familiarization trials with feedback with the four ankle inversion landing positions numbered 1 through 4 in ascending order, followed by 40 testing trials without feedback, delivered in random order. Each examination session was separated by a two-minute rest interval.

### Statistical Analysis

The mean and standard deviation were used to summarize the quantitative data. A two-by-two factorial analysis of variance (ANOVA) was conducted to examine the effects of different types of insoles on ankle proprioceptive ability, specifically the ankle inversion discrimination scores, and lower limb muscle activity in soccer players with and without CAI. This analysis also investigated the factor interactions during drop-landing tasks on the AIDAL. The main effects of the participants' ankle conditions and the type of insoles on AIDAL scores and muscle activity, as well as the interactions between these factors were evaluated. The level of significance was set at *p* ≤ 0.05. All statistical tests were performed using IBM SPSS Statistical Software version 25 for Windows (Chicago, IL, USA).

## Results

Thirty-two soccer players participated in this study, 16 without CAI and 16 with CAI. Participants’ demographic data and CAIT scores can be found in Table 1. No statistical differences between groups were found for age (*p* = 0.71), body height (*p* = 0.95), body mass (*p* = 0.81), and years of sport specific training (*p* = 0.16). Soccer players with CAI reported significantly lower CAIT scores (*p* < 0.001).

**Table 1:** Demographic and Cumberland Ankle Instability Tool (CAIT) data of the participants in the non-chronic ankle instability (non-CAI) and chronic ankle instability (CAI) groups. Data are reported as mean (SD).

Variables	non-CAI (n = 16)	CAI (n = 16)	*p* value
Age (years)	20.3 (0.86)	20.1 (1.03)	0.71
Body height (cm)	171.5 (6.70)	171.6 (5.16)	0.95
Body mass (kg)	66.7 (8.55)	65.9 (10.5)	0.81
Training experience (years)	6.18 (1.29)	6.82 (1.33)	0.16
CAIT scores	26.6 (2.16)	21.1 (1.63)	< 0.001^*^

* = statistically significant difference

**Figure 3:**
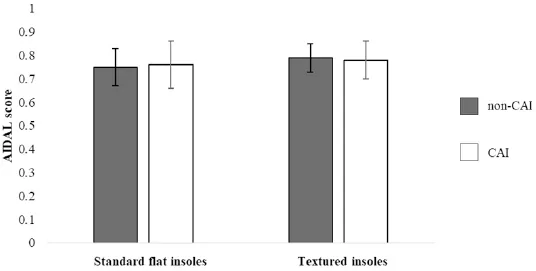
Participant’s AIDAL score.

Figure 3 and Table 2 show the AIDAL scores of each group, main effects and interactions of ankle and insole conditions on AIDAL scores, respectively. There were no statistically significant differences in the main effects of ankle and insole conditions, nor were their interactions significant.

**Table 2:** Main effect and interactions of ankle and insole conditions on the AIDAL score.

	Main effect: ankle conditions			Main effect: insole conditions			Interaction		
	F	*p*	Ƞp^2^	F	*p*	Ƞp^2^	F	*p*	Ƞp^2^
AIDAL score	(1,60) 2.09	0.15	0.034	(1,60) 0.002	0.96	< 0.001	(1,60) 0.73	0.40	0.012

**Figure 4:**
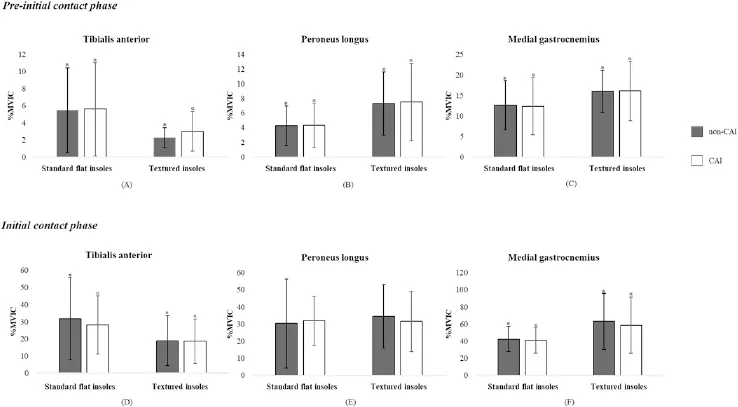
Normalized muscle activity (%MVICs) during the pre-initial contact phase: (A) tibialis anterior, (B) peroneus longus, and (C) medial gastrocnemius; and during the initial contact phase: (D) tibialis anterior, (E) peroneus longus, and (F) medial gastrocnemius during drop-landing on the AIDAL. * = significant difference between insoles in the non-CAI group, a = significant difference between insoles in the CAI group

Figure 4 and Table 3 illustrate normalized muscle activity (%MVICs), main effects and interactions of ankle and insole conditions during pre-initial contact and initial contact phases on %MVIC, respectively. A two-by-two factorial ANOVA was conducted to assess the main effects and interactions of ankle and insole conditions on %MVICs of TA, MG, and PL muscles. During the pre-initial contact phase, the non-CAI group had significantly higher TA activity (M = 5.53; SD = 5.15) than the CAI group (M = 2.62; SD = 1.86), F(1,60) = 8.78, *p* = 0.04, Ƞp^2^ = 0.13. In contrast, the CAI group showed significantly higher PL activity (M = 7.39; SD = 4.75) than the non-CAI group (M = 4.28; SD = 2.83), F(1,60) = 9.83, *p* = 0.003, $\eta_{p^{2}}$ = 0.14. Moreover, it was found that the CAI group had greater MG activity (M = 16.0; SD = 6.15) than the non-CAI group (M = 12.5; SD = 6.38), F(1,58) = 4.77, *p* = 0.03, Ƞp^2^ = 0.08. However, no statistically different main effects of the insole conditions or interactions with the ankle conditions on %MVIC of any muscles were discovered during the pre-initial contact phase.

**Table 3:** Main effects and interactions of ankle and insole conditions on %MVIC during pre-initial contact and initial contact of drop-landing on the AIDAL.

	Main effect: ankle conditions			Main effect: insole conditions			Interaction		
F	*p*	Ƞp^2^	F	*p*	Ƞp^2^	F	*p*	Ƞp^2^
*Pre-initial contact*									
Tibialis anterior	(1,60) 8.78	0.04*	0.13	(1,60) 0.21	0.65	0.004	(1,60) 0.097	0.76	0.002
Peroneus longus	(1,60) 9.83	0.003*	0.14	(1,60) 0.02	0.90	< 0.001	(1,60) 0.002	0.96	< 0.001
Medial gastrocnemius	(1,58) 4.77	0.03*	0.08	(1,58) 0.002	0.97	< 0.001	(1,58) 0.015	0.90	< 0.001
*Initial contact*									
Tibialis anterior	(1,60) 6.46	0.01*	0.10	(1,60) 0.21	0.65	0.004	(1,60) 0.14	0.71	0.002
Peroneus longus	(1,60) 0.09	0.76	0.002	(1,60) 0.14	0.91	< 0.001	(1,60) 0.14	0.71	0.002
Medial gastrocnemius	(1,59) 8.96	0.004*	0.13	(1,59) 0.20	0.66	0.003	(1,59) 0.04	0.84	0.001

* = statistically significant difference

During the initial contact phase, the non-CAI group demonstrated significantly higher TA activity (M = 29.9; SD =20.6) than the CAI group (M = 18.7; SD = 13.6), F(1,60) = 6.46, *p* = 0.01, Ƞp^2^ = 0.1. However, they had significantly lower MG activity (M = 41.5; SD = 14.8) than the CAI group (M = 60.8; SD = 32.5), F(1,59) = 8.96, *p* = 0.004, Ƞp^2^ = 0.13. Additionally, no main effects of the insole conditions or interactions with the ankle conditions were found in this phase.

## Discussion

This study aimed to investigate the effects of wearing different types of insoles on lower limb muscle activity and ankle proprioceptive ability during drop-landing tasks in soccer players with and without CAI. The findings indicate that CAI soccer players exhibited a different ankle strategy involving alteration in ankle muscle activity, regardless of the insole type. Despite these differences in muscle activity, soccer players with and without CAI had comparable ankle proprioceptive performance with both types of insoles. Furthermore, the current study showed that following the application of both types of insoles, there was a comparable level of ankle muscle activity between CAI and non-CAI soccer players.

The primary finding of this study is that CAI soccer players demonstrated altered lower limb muscle activity compared to non-CAI soccer players during drop-landing tasks on the AIDAL, regardless of the insole worn. Both pre-activation and reflexive responses were affected. During the pre-initial contact phase of the drop-landing tasks, there was an increase in PL activity in the CAI group. Our results are consistent with previous research, which found that individuals with functional ankle instability and/or CAI exhibited greater levels of anticipatory PL activity during the pre-initial contact (Delahunt et al., 2006a; Webster et al., 2016). Following a recurrence of ankle inversion injuries in individuals with CAI, an alteration in feed-forward motor control may explain the increased activity of the PL. To avoid an inverted ankle position, individuals with CAI seem to compensate in their landing strategy by enhancing the anticipatory activity of the PL, aiming to evert the ankle before the foot contact with the ground (Webster et al., 2016). This strategy differs from that of individuals without CAI, indicating a unique protective mechanism. Thus, the assumptions drawn from previous studies find substantial support in our findings.

However, the current findings appear to contradict those of previous studies (Suda et al., 2009; Tretriluxana et al., 2021). Those researchers detected a decrease in anticipatory PL activity following a drop-jump task in individuals with ankle instability. This reduction in PL activity may suggest a more inverted ankle posture in those with CAI compared to the control group, indicating a compromised neuromuscular response that could exacerbate the risk of ankle sprains. Several methodological differences between the mentioned studies and the current study may account for the different results. First, previous research emphasized single-leg landings (Suda et al., 2009; Tretriluxana et al., 2021), whereas our study was conducted on double-leg landings. Due to the contribution of the contralateral leg in maintaining stability during landings, the requirements for these two tasks are quite different. Secondly, previous studies did not incorporate any perturbations during the landing tasks; participants were merely instructed to land on the level ground following jumps. In contrast, our study required participants to land on a tilt platform as part of the AIDAL, designed to replicate the mechanism of an ankle sprain. This added perturbation likely influenced neuromuscular strategies, as it is possible that CAI individuals employed a protective strategy by increasing the preparatory activity of the PL muscle during testing to prevent ankle inversion.

In addition, the present study found that CAI soccer players exhibited less-activation of the TA and over-activation of the MG during both the pre-initial and initial contact phases of the drop-landing tasks, suggesting that the ankles of the CAI group were in a plantarflexed position during these tasks. This imbalance suggests that the ankles of the CAI group were in a more plantarflexed position during these tasks, which is associated with a higher incidence of recurrent ankle sprains. In contrast, non-CAI soccer players demonstrated increased TA activity, suggesting a different ankle protective mechanism, as noted in a previous study (Allet et al., 2017). Higher TA activity promotes ankle dorsiflexion, moving the ankle joint into a more stable, close-packed position, thereby providing additional protection to the lateral ligament complex. The absence of this movement strategy in CAI players leads to excessive ankle plantarflexion during drop-landing tasks, increasing the risk of ankle sprains (Labanca et al., 2021). However, it should be noted that increased preparatory TA activity during the pre-initial contact phase could result in a more inverted ankle position before the ground contact, a pattern previously observed in individuals with functional ankle instability (Caulfield et al., 2004; Delahunt et al., 2006b; Suda et al., 2009). Since the TA functions as both an inverter and a dorsiflexor of the ankle, this strategy presents a paradox. It is possible that the non-CAI group adopted a different strategy by landing with their ankle in a less plantarflexed position as a precautionary strategy to preserve ankle stability. This strategy might shift the impact absorption to adjacent joints, such as the knee or the hip, thereby preserving ankle stability. However, it is important to note that this study did not measure kinematic variables. Future research should include the measurement of these variables to provide a more comprehensive understanding of the mechanisms underlying these findings.

Despite the observed differences in ankle muscle activity during drop-landing tasks, the present study found no significant differences in ankle proprioceptive ability between CAI and non-CAI soccer players. This finding contrasts with previous research, which has consistently reported that individuals with CAI exhibit deficient ankle proprioceptive abilities compared to non-CAI counterparts (Xue et al., 2021). Several factors may account for these differences.

Firstly, previous studies typically tested ankle proprioceptive abilities under barefoot conditions, allowing for direct interaction with the testing surface and more accurate proprioceptive feedback (Han et al., 2021, 2022; Steinberg et al., 2016b). In contrast, the present study tested participants while they were wearing soccer footwear, which may have dampened the sensory feedback from the foot, potentially obscuring the true proprioceptive performance of the participants. By wearing footwear, the tactile input and mechanoreceptor stimulation on the plantar surface of the foot could be reduced, leading to similar proprioceptive performance between CAI and non-CAI soccer players regardless of any inherent proprioceptive differences.

Secondly, individuals with CAI often develop adaptive strategies to cope with the inherent instability of their ankle joints (Hertel and Corbett, 2019). Over time, their proprioceptive system may improve, allowing them to better detect and respond to small changes in ankle positioning. This adaptive mechanism serves as a compensatory response to address structural instability. The increased recognition of the joint position might be seen as an enhancement in proprioceptive ability. Furthermore, individuals with CAI may increasingly depend on proprioception as a primary source of feedback due to their joint instability. Relying more on this information may enhance sensory processing and integration, leading to better proprioceptive ability.

In addition, the absence of controlled rehabilitation protocols or soccer training routines in this study might have impacted the outcomes. It is plausible that CAI soccer players engage in various individualized procedures to address their condition, such as specific strength training, balance exercises, or proprioceptive training regimens. These activities may stimulate proprioceptive pathways, enhancing the brain's ability to accurately interpret sensory information (Fakontis et al., 2023). Over time, such interventions can lead to adaptations in the CNS that compensate for proprioceptive deficits, resulting in similar proprioceptive abilities between soccer players with and without CAI.

Lastly, the brief exposure time to the textured insoles during drop-landing tasks in the current study may have contributed to the lack of significant differences. Previous studies have shown the benefits of textured insoles in less dynamic activities, such as a star excursion balance test or a standing balance test, where exposure times are much longer (Abbasi et al., 2019; Steinberg et al., 2016a). Extended duration of exposure provides continuous sensory input and increased activation of mechanoreceptors, resulting in significant enhancements in proprioceptive performance and neuromuscular control. The brief length of contact with the insoles during the dynamic drop-landing task restricts the amount of time for sensory input. This may not have been sufficient to provide the same level of neuromuscular adaptation and proprioceptive enhancement as shown in activities that are more stationary. As a result, the potential benefits of insoles might not become apparent in such a brief and dynamic context.

The present study revealed no significant difference in ankle proprioceptive ability and ankle muscle activity following the application of both types of insoles. These results contrast with previous research, which has demonstrated positive effects of textured insoles on improving ankle proprioceptive ability (Palazzo et al., 2022; Robb et al., 2022; Steinberg et al., 2016a) and on muscle activation onset times of the ankle muscles (Dingenen et al., 2015). Several factors could account for these differences. Firstly, the present study tested ankle proprioceptive ability of the participants during drop-landing on an inverted platform of the AIDAL, a more challenging task compared to the standing or walking conditions used in previous studies. This increased complexity may have masked the potential benefits of the insoles on proprioception and muscle activity. Secondly, all participants were asked to wear stockings and soccer footwear during the insole application, which may have diminished the sensory feedback from the foot, thereby hindering proprioceptive performance and muscle activity. This layer of material could obscure the tactile stimuli provided by the insoles, reducing their effectiveness. In addition, previous research typically evaluated the effects of insoles over a longer period, whereas the present study focused on the immediate effects of insole application. The long-term use of insoles might allow for neuromuscular adaptations that enhance proprioception and muscle activation (Hatton et al., 2016; Lirani-Silva et al., 2017; Steinberg et al., 2015), which were not captured in the short-term assessment of the current study. These methodological differences highlight the importance of considering task difficulty and duration of insole use when interpreting the effectiveness of insole interventions for individuals with CAI.

## Limitations

The study has several limitations that warrant consideration. Firstly, the assessment focused solely on the immediate effects of insole application, potentially overlooking long-term impacts that could lead to neuromuscular adaptations enhancing proprioception and muscle activation. Secondly, participants wore stockings and soccer footwear during testing, which likely diminished sensory feedback from the insoles, possibly reducing their effectiveness. Finally, this study did not control for the participants’ previous exposure to rehabilitation protocols or specific training routines, which could have confounded proprioceptive abilities and muscle activity, highlighting the need for a more controlled approach to isolate the effects of the insoles and provide clearer insights into their efficacy in future studies.

## Conclusions

This study found that different types of insoles had no significant immediate effects on lower limb muscle activity in soccer players with and without CAI during drop-landing tasks. Regardless of the insole type, CAI players exhibited increased PL activity during the pre-initial contact, which seems to be a compensating strategy adopted by CAI soccer players to enhance ankle stability. Decreased TA activation and increased MG activity during pre-initial and initial contact phases may contribute to the high incidence of recurrent ankle sprains observed in CAI individuals and this may inform targeted rehabilitation programs. The lack of immediate effects from insoles on muscle activity suggests that their influence on sensory input and foot positioning may require prolonged use to induce measurable neuromuscular adaptations. Future research should investigate the long-term application of insoles to determine their impact on proprioception, neuromuscular control, and injury prevention in soccer players with CAI.
